# Working Memory Load Reduces the Electrocortical Processing of Negative Pictures but Accentuates Their Processing at Subsequent Viewing

**DOI:** 10.1111/psyp.70398

**Published:** 2026-09-03

**Authors:** Ha Jeong Park, T. Bryan Jackson, Annmarie MacNamara

**Affiliations:** ^1^ Department of Psychological and Brain Sciences Texas A&M University College Station Texas USA; ^2^ Vanderbilt Memory and Alzheimer's Center Vanderbilt Health Nashville Tennessee USA; ^3^ Department of Psychiatry and Behavioral Sciences Texas A&M University College Station Texas USA; ^4^ Institute for Neuroscience Texas A&M University College Station Texas USA

**Keywords:** emotion processing, event‐related potentials, late positive potential, recognition, working memory load

## Abstract

Cognitive load can reduce attention to negative stimuli, but its utility as an emotion regulation strategy may depend on whether it functions like avoidance, leading to short‐term reductions in stimulus salience that nonetheless preserve or even heighten response to stimuli at subsequent encounters. Here, 73 participants performed a working memory task interspersed with negative and neutral pictures, prior to viewing the same pictures again alongside new negative and neutral pictures in a surprise recognition task. During the first task, working memory load reduced picture processing, replicating prior findings. During the second task, negative pictures that had previously been presented under high‐load and new negative pictures were processed similarly, receiving more attentional resources than negative pictures that had been presented under low‐load. Subjective picture ratings showed similar results, with negative pictures that had previously been presented under high‐load rated as more arousing than those that had been presented under low‐load, and indistinguishable from new negative pictures. Negative pictures that had previously been presented on high‐load compared to low‐load trials also had lower recognition accuracy. The overall direction of effects differed for neutral compared to negative pictures, but pictures that had been presented under high working memory resembled new pictures in the responses they elicited across both picture types. For negative stimuli, results suggest a mechanism through which disengaged emotion regulation strategies may fail to attenuate and may even increase emotional responses to stimuli when encountered again outside of the initial regulation context.

## Impact Statement

Working memory load—a disengaged emotion regulation strategy—reduced the LPP elicited by pictures. During a subsequent recognition task, negative pictures originally presented under high (versus low) working memory load were remembered less well, rated as more arousing, and elicited electrocortical responses resembling those elicited by new negative pictures. Therefore, disengaged emotion regulation strategies may interfere with stimulus encoding, preventing reductions in stimulus processing that would otherwise occur with repeated exposure.

## 1 Introduction

Salient stimuli capture attention, even when task‐irrelevant (e.g., Hickey et al. 2006). This is particularly true for emotional stimuli (Smith et al. 2004). Importantly, this bottom‐up, stimulus‐driven attention can be balanced by top‐down, goal‐directed attention (Corbetta and Shulman 2002; Van Der Burg et al. 2007). For instance, both distraction (e.g., Adamczyk et al. 2023) and working memory load (Barley et al. 2021; MacNamara, Ferri, et al. 2011), lead to rapid reductions in subjective emotional and electrocortical response to negative pictures. Distraction involves intentionally focusing attention on content other than the emotional stimulus. On the other hand, working memory tasks do not involve willful re‐direction of attention, but engage a limited‐capacity system responsible for maintaining and manipulating task‐relevant information (Baddeley 2003; Cowan 2010; Goldman‐Rakic 1995). Therefore, cognitive load, such as throwing oneself into a task at work or even performing difficult arithmetic has been suggested as a means of regulating emotion (Braunstein et al. 2017; Kanske et al. 2011; Van Dillen and Koole 2007). Together, negative emotion downregulation strategies that minimize processing of stimulus content are referred to as disengaged (versus engaged) strategies (MacNamara 2025; Sheppes et al. 2011). Although disengaged emotion regulation strategies are effective in the short‐term, they may *increase* the processing of emotional stimuli in the longer term (Jiang et al. 2020; Paul et al. 2016; Thiruchselvam et al. 2011), calling into question their longer term utility (MacNamara 2025).

For example, Paul et al. (2016) examined the lasting effects of a disengaged emotion regulation strategy, distraction, on the late positive potential (LPP), a centroparietally maximal event‐related potential (ERP) that begins approximately 300 ms after stimulus onset and is larger for emotional compared to neutral stimuli (Cuthbert et al. 2000; Hajcak and Olvet 2008; Pastor et al. 2008). Participants were asked to either attend to or distract themselves from negative and neutral pictures prior to performing a passive picture viewing task with the same stimuli. Results showed that pictures that had previously been presented in the distract condition elicited larger LPPs than pictures that had been presented in the attend condition, suggesting reduced habituation due to more superficial processing of pictures in the distract condition. Likewise, Thiruchselvam et al. (2011) found that the LPP elicited by negative pictures originally presented under instructions to distract was larger than that elicited by pictures that had been passively viewed or reappraised. Jiang et al. (2020) extended these results to find that whereas distraction may successfully reduce attention to previously encountered unpleasant stimuli after a short interval (i.e., 5 min), its effects reversed after a longer interval (i.e., 30 min). These results suggest that in spite of effective and rapid reduction of emotional stimulus processing in the short‐term (e.g., Sheppes et al. 2014), disengaged emotion regulation strategies may be less effective in the long term (MacNamara 2025).

In our prior work using working memory load to reduce picture processing, participants viewed six or two letters followed by a task‐irrelevant emotional or neutral picture. At the end of each trial, participants were asked to recall the letters presented at the beginning of the trial. Results showed that pictures presented under high‐load (six letters) compared to low‐load (two letters) elicited smaller LPPs, indicating that participants allocated fewer processing resources toward task‐irrelevant stimuli when task demands were high (Barley et al. 2021; Cheng et al. 2022; MacNamara et al. 2011). Similar effects have been observed in work from other groups as measured using the LPP (e.g., Adamczyk et al. 2022; Gan et al. 2017) and amygdala activity elicited by negative stimuli (Van Dillen et al. 2009). Complementing the literature on distraction, these studies indicate that even in the absence of willful attempts to downregulate emotion, disengagement via a cognitively demanding task can reduce the salience of emotional stimuli. Nonetheless, like other disengaged emotion regulation strategies, working memory load might also fail to exert longer‐lasting effects on stimulus processing, and could even lead to increased processing of emotional stimuli when encountered later on.

In addition to being ineffective at reducing emotional response to stimuli over the longer term, disengaged emotion regulation strategies might also impair memory for these stimuli. That is, if stimuli are processed only superficially at first encounter, this may interfere with encoding of these stimuli in longer term memory. Indeed, encoding failures might explain in part why disengaged emotion regulation strategies fail to exert lasting effects on the emotional salience of stimuli—that is, at subsequent encounter, these stimuli might be processed as if, to some extent, they were never seen before.

A parietally‐maximal ERP component referred to as the “old/new effect” (Wilding and Ranganath 2011) is a neural marker in memory research that distinguishes between stimuli that have been seen before and novel stimuli (Rugg et al. 1996). Though there is debate as to the functional significance of this component, more positive amplitudes for old versus new stimuli between approximately 300–800 ms poststimulus onset may reflect item familiarity followed by recollection during recognition tasks (Rugg et al. 1996). There is also evidence for a later, negative‐going component, the late posterior negativity (LPN), that emerges around 800 ms post‐stimulus onset at parietal sites and is larger (i.e., more negative) for old compared to new stimuli (Johansson and Mecklinger 2003; Mecklinger et al. 2016)—that is, a reversal effect (Rugg et al. 1996). More elaborate or effortful retrieval, such as recalling detailed features of prior stimuli (e.g., color in addition to object identity) is associated with a more negative LPN (Cycowicz et al. 2001). Thus, the LPN has been interpreted as an index of action monitoring, or how carefully an individual evaluates and verifies their retrieval process. In this sense, the negative‐going LPN may be a suppressor of the concurrent, positive‐going LPP, which is sensitive to motivational salience. In other words, during a recognition task, increased retrieval monitoring (i.e., a more negative LPN) may attenuate the overall positivity of the ERP waveform, resulting in a reduced LPP for previously encoded stimuli. In contrast, when a stimulus is processed as novel, possibly due to insufficient encoding, reduced engagement in retrieval monitoring may yield a smaller (i.e., less negative) LPN, allowing the LPP to remain relatively more positive. Therefore, investigating both the old/new effect and the LPP during the entirety of picture presentation duration may inform understanding of whether working memory load affects the initial recognition and more elaborated processing of these pictures.

Here, we assessed how working memory load would affect the electrocortical processing, emotional salience, and recognition of negative and neutral pictures when encountered later on. To this end, participants performed a version of the working memory task used in our prior work, in which they viewed negative and neutral pictures presented under high and low working memory load prior to rating each picture on valence and arousal (MacNamara et al. 2011, 2019; MacNamara and Proudfit 2014). Immediately following this, participants performed a surprise recognition task, in which they viewed the same negative and neutral pictures as they had seen before, as well as new negative and neutral pictures. For each picture, participants were asked to indicate whether they had seen it before (“Old”) or had not seen it before (“New”) prior to rating each picture on valence and arousal. EEG was recorded throughout both tasks.

In the working memory task, we expected that working memory load would reduce the LPP, as in our prior work (e.g., MacNamara, Ferri, et al. 2011; MacNamara and Proudfit 2014). In the recognition task, we expected that early parietal positivities (400–800 ms) would be larger for old compared to new pictures (Rugg et al. 1996; Smith et al. 2004; Weymar et al. 2011) but that this effect would be smaller for pictures previously presented under high compared to low working memory load. This hypothesis was based on the notion that pictures initially presented under high‐load would have been processed less deeply and would therefore be perceived as less familiar (more like new pictures) at subsequent encounters; for this reason, we also thought that recognition accuracy would be worse and emotional salience higher for pictures presented under high‐load. During later portions of picture processing (i.e., 800–1400 and 1400–2000 ms post picture onset), we thought that pictures originally presented under high working memory load would elicit *larger* LPPs than those previously presented under low working memory load, because they would resemble novel pictures, which receive more processing than old pictures during later stages (Ferrari et al. 2017; Paul et al. 2016; Weymar et al. 2011). If these hypotheses were to be confirmed, it would indicate that although working memory load might reduce picture salience at initial encounter, it might lead to less desirable long‐term effects—that is, *increased* processing, worse memory and greater emotional salience of pictures when pictures are subsequently encountered.

## 2 Method

### 2.1 Participants

The final sample consisted of 73 participants (48 females, 25 males) between the ages of 18 and 23 years (*M* = 18.9 years, SD = 1.17), who completed the experiment for course credit. An additional six individuals participated in the experiment but were excluded—two were excluded due to technical difficulties, one did not finish the task, and three were outliers in terms of behavioral performance on the recognition task, according to Grubbs' test for outliers (Grubbs 1969). Subjective picture rating data for the recognition task was also missing for one participant. Study procedures were in compliance with the Helsinki Declaration of 1975 (as revised in 1983) and were approved by the Texas A&M institutional review board.

### 2.2 Materials

Across both tasks, participants viewed a total of 120 negative pictures (e.g., threatening animals, people with guns) and 120 neutral pictures (e.g., household objects, neutral faces) from the International Affective Picture System (Lang et al. 2005) and Emotional Picture Set (Wessa et al. 2010).^1^ Half of these pictures (60 negative; 60 neutral) were shown to participants under high or low working memory load during the working memory load task; these were counterbalanced across participants.^2^ The remaining pictures (60 negative; 60 neutral) served as new stimuli in the recognition task, in which participants also viewed the “Old” pictures that had previously been presented under either high or low working memory load. In both tasks, participants used the Self‐Assessment Manikin 9‐point scales (Bradley and Lang 1994) to make valence and arousal ratings for each picture, with higher numbers indicating higher arousal and more pleasant valence.

### 2.3 Procedure

**FIGURE 1:**
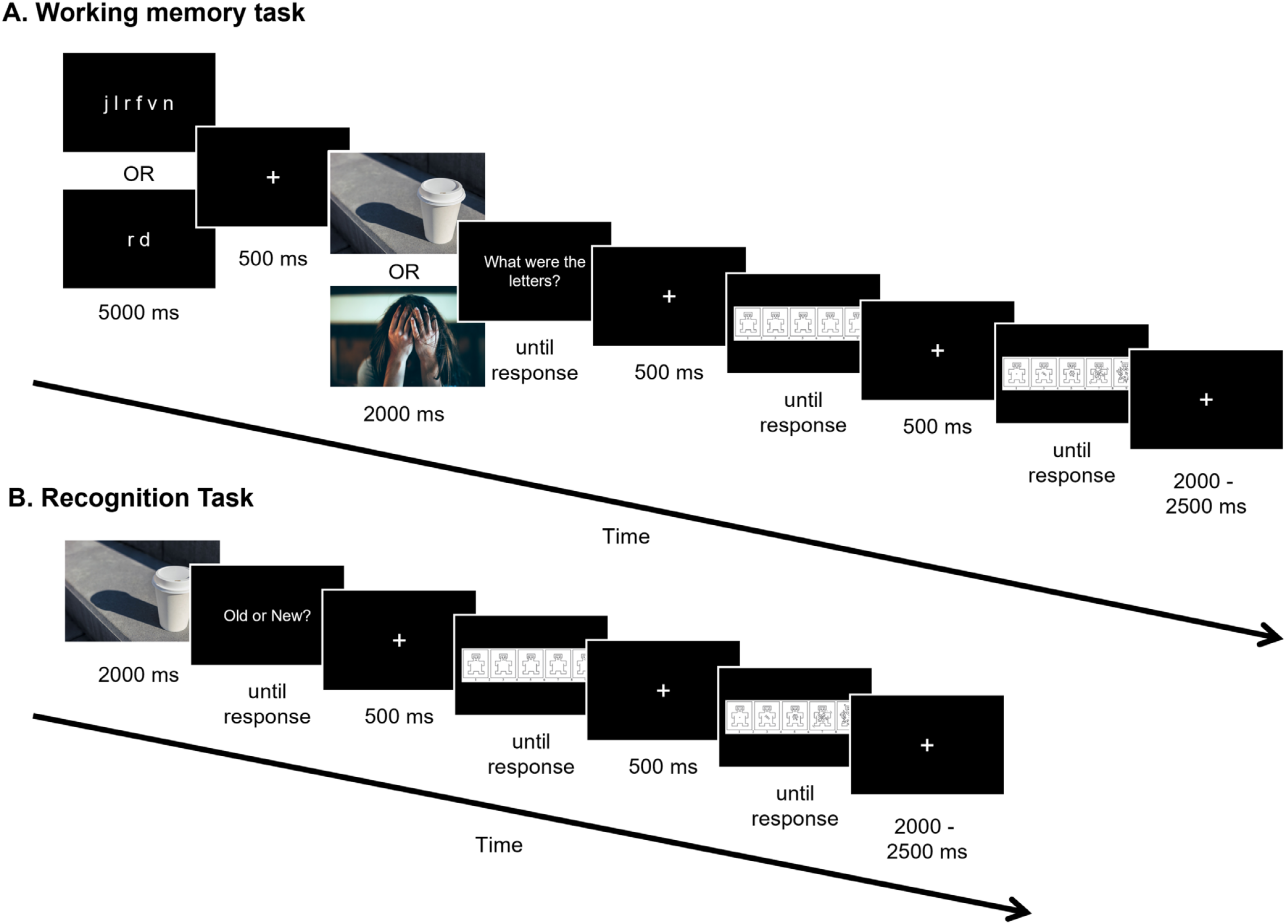
Depiction of the working memory and recognition tasks. (A) During the working memory task, participants were asked to remember a string of letters (six letters/high working memory load or two letters/low working memory load) which were followed by either a negative or neutral picture. Participants were then asked to recall the string of letters they saw prior to picture presentation. After their response participants were asked to rate the valence and arousal of the picture that they saw. (B) During the recognition task, participants viewed the same pictures from the working memory task as well as new negative and neutral pictures. After picture offset, participants indicated whether the picture was “Old” or “New”, followed by rating picture valence and arousal.

Upon arrival, participants were consented. Next, participants performed a working memory task interspersed with the presentation of 60 negative and 60 neutral, task‐irrelevant images, followed by a surprise recognition task, in which they viewed all 120 pictures from the working memory task, in addition to 60 negative and 60 neutral new pictures. Participants completed the recognition task immediately after the working memory task, with a short break (~5 min) in between for instructions. In total, the experiment took approximately 1 h but varied due to differences in response time and break length. The two tasks are depicted in Figure 1.

### 2.4 Working Memory Task

Participants performed a variant of the working memory load task described in our prior work (Barley et al. 2021; Cheng et al. 2022; MacNamara et al. 2011, 2019). Each trial began with the presentation of a two‐ or six‐letter string for 5000 ms, followed by a neutral or negative picture presented for 2000 ms. Participants were asked to remember the letters and enter them at the end of each trial, prior to rating the valence and arousal of each picture. Participants viewed 120 pictures in total, resulting in the following trial types: 30 neutral pictures presented under low‐load (low‐load neutral), 30 neutral pictures presented under high‐load (high‐load neutral), 30 negative pictures presented under low‐load (low‐load negative), and 30 negative pictures presented under high‐load (high‐load negative). Behavioral responses (letter recall and subjective ratings) were self‐paced, and the trial ended when all responses had been made. During the intertrial interval, a white fixation cross was displayed on a black background for 2000–2500 ms. Trials were presented in a random order, and participants received a break halfway through the task. Participants completed four practice trials (two high‐load and two low‐load trials in random order) prior to beginning the real task. Feedback for correct or incorrect answers was not given during the practice trials or in the real task.

### 2.5 Recognition Task

Following the working memory task, participants performed a surprise recognition task in which they viewed pictures from the working memory task and new pictures, resulting in 6 trial types: 30 neutral pictures previously presented under low‐load (low‐load neutral), 30 neutral pictures previously presented under high‐load (high‐load neutral), 30 negative pictures previously presented under low‐load (low‐load negative), 30 negative pictures previously presented under high‐load (high‐load negative), 60 neutral pictures not previously presented (new neutral) and 60 negative pictures not previously presented (new negative). Participants viewed each picture for 2000 ms. Following picture offset, participants used the left or right mouse button to indicate whether the picture was “Old” (they recognized the picture from the previous task) or “New” (they had not seen the picture before). Next, they rated the picture on valence and arousal. Responses (old/new and subjective ratings) were self‐paced and the trial ended when all responses had been made. During the inter‐trial interval, a white fixation cross was displayed on a black background for 2000–2500 ms. Trials were presented in a random order and participants received a break between every 20 trials. Participants completed four practice trials using pictures that were not included in the working memory task prior to beginning the real task. Feedback for correct or incorrect answers was not given during the practice trials or in the real task.

### 2.6 EEG Recording and Data Reduction

Continuous EEG was recorded using an ActiCap and the ActiCHamp amplifier system (Brain Products, Munich, Germany). Thirty‐two electrode sites were used based on the 10/20 system. The electrooculogram (EOG) was recorded from four facial electrodes: two that were placed approximately 1 cm above and below the right eye, forming a bipolar channel to measure vertical eye movement and blinks and two that were placed approximately 1 cm beyond the outer edges of each eye, forming a bipolar channel to measure horizontal eye movements. The EEG data were digitized at 24‐bit resolution and a sampling rate of 1000 Hz, in keeping with our prior work (Barley et al. 2021; Cheng et al. 2023). During EEG acquisition, impedance was maintained below 30 kΩ.

EEG data were processed offline using Brain Vision Analyzer 2 software (Brain Products, v2.1.2). For both the working memory task and the recognition task, signal from each electrode was re‐referenced to the average of the left and right mastoids (TP9/10). A zero‐phase‐shift Butterworth band‐pass filter was applied to continuous data, with a 2nd‐order high‐pass component (0.01 Hz; 12 dB/octave roll‐off) and a 4th‐order low‐pass component (30 Hz; 24 dB/octave roll‐off). Data were then segmented for each trial beginning 200 ms prior to picture onset and continuing for 2200 ms (i.e., until picture offset). Eye blink and ocular corrections used the method developed by Miller et al. (1988). Artifact analysis was used to identify a voltage step of more than 50.0 μV between sample points, a voltage difference of 300.0 μV within a trial, and a maximum voltage difference of less than 0.50 μV within 100 ms intervals. As with our prior work, trials were also inspected visually for any remaining artifacts, and data from individual channels containing artifacts were rejected on a trial‐to‐trial basis (Barley et al. 2021; Cheng et al. 2022; MacNamara et al. 2019). A minimum of 50% of trials per condition was required for participant inclusion in the ERP analysis.^3^ Finally, baseline correction for each trial was performed using the 200 ms prior to picture onset.

Centroparietal amplitudes were scored separately for each task at a pooling of Cz, Pz, CP1, and CP2, between 400 and 800 ms (Codispoti et al. 2006; Curran and Friedman 2004), 800–1400 ms (Rugg et al. 1996) and 1400–2000 ms (MacNamara et al. 2011; MacNamara and Proudfit 2014) following picture onset. To facilitate comparison between the working memory and recognition tasks, we used these same time windows in both sets of tasks. All time windows corresponded to the LPP for the working memory task. In the recognition task, the 400–800 ms time window referred to the Old‐New effect (Rugg et al. 1996), whereas both the 800–1400 and 1400–2000 ms time windows captured the summation of the LPP and LPN, in line with the broader ERP recognition literature (Mecklinger et al. 2016). In line with prior work, ERPs elicited during the recognition task were scored only for correctly classified old and new pictures (Herron et al. 2003; Ventura‐Bort et al. 2016).

### 2.7 Picture Ratings

For each task, subjective valence and arousal ratings were averaged for each condition. In line with the ERP analyses, only ratings on correct trials were analyzed for the recognition task (Ventura‐Bort et al. 2016).

### 2.8 Behavioral Data

Responses to the working memory task were considered correct if they contained the same letters as those that had been presented at the beginning of the trial, entered in the same order in which they had originally been presented (MacNamara et al. 2011; MacNamara and Proudfit 2014). The percentage of correct responses per condition was calculated (i.e., for each of low‐load neutral, high‐load neutral, low‐load negative, high‐load negative).

For the recognition task, the percentage of correctly classified items (i.e., as “Old” or “New”) was calculated separately for each condition (i.e., low‐load neutral, high‐load neutral, low‐load negative, high‐load negative, new neutral and new negative). In keeping with prior work, reaction time on the recognition task (for correct trials only) was also assessed (Rugg et al. 1996).

### 2.9 Data Analyses

For the working memory task, a 2 (condition: low‐load, high‐load) × 2 (picture type: neutral, negative) repeated‐measures analysis of variance (ANOVA) was performed separately for ERPs in each time window, subjective ratings, and accuracy. For the recognition task, a 3 (condition: low‐load, high‐load, new) × 2 (picture type: neutral, negative) repeated‐measures ANOVA was performed separately for ERPs in each time window, subjective ratings, accuracy, and reaction time data.^4^ Follow‐up ANOVAs and paired *t‐*tests were used as appropriate to examine effects that were significant at the omnibus level. Benjamini–Hochberg correction (Benjamini and Hochberg 1995) was applied to correct for false discovery rate as needed. Statistical significance was adjusted using Greenhouse–Geisser correction applied to *p*‐values for violations of sphericity, as necessary. All analyses were performed using SPSS statistical software version 25 (IBM, Armonk, NY, v25.0).

## 3 Results

Table 1 presents means and standard deviations for ERPs, ratings, accuracy, and reaction time, shown separately for each task, condition, and picture type. The working memory task included four trial types: low‐load neutral, low‐load negative, high‐load neutral, and high‐load negative. The recognition task included the same trial types for previously presented pictures, with two additional trial types for pictures that have not been presented during the working memory task (new neutral and new negative).

**TABLE 1:** Means (and standard deviations) for all dependent variables, shown separately for each task, picture type and condition.

Task	Working memory				Recognition					
Picture type	Neutral		Negative		Neutral			Negative		
Condition	Low	High	Low	High	Low	High	New	Low	High	New
LPP; Old/New (μv; 400–800 ms)	0.84 (4.98)	−2.10 (4.68)	4.80 (6.19)	1.51 (5.91)	3.34 (3.46)	3.47 (4.75)	1.79 (4.32)	6.26 (4.63)	6.66 (5.24)	5.41 (5.06)
LPP (μv; 800–1400 ms)	3.76 (4.61)	0.17 (4.06)	8.03 (5.85)	5.16 (5.65)	5.22^a,b^ (3.31)	4.68^a,b^ (4.23)	4.20^a^ (3.47)	8.00^a^ (4.40)	9.00^a,b^ (4.79)	9.21^b^ (4.69)
LPP (μv; 1400–2000 ms)	4.72^a^ (5.26)	0.04^b^ (4.67)	8.16^a^ (6.55)	5.22^b^ (6.23)	3.69 (3.99)	3.13 (4.05)	4.44 (3.61)	6.40 (4.79)	6.98 (4.90)	8.42 (4.51)
Valence ratings	5.17 (0.56)	5.20 (0.58)	2.76 (0.76)	2.76 (0.80)	5.06^a,b^ (0.48)	5.12^a,b^ (0.55)	5.16^a^ (0.54)	2.83^a^ (0.89)	2.79^a^ (0.90)	2.81^a^ (0.85)
Arousal ratings	1.67 (0.64)	1.63 (0.67)	4.67 (1.68)	4.67 (1.67)	1.56^a^ (0.70)	1.51^b^ (0.70)	1.49^b^ (0.68)	4.54^a^ (1.92)	4.71^b^ (1.94)	4.68^b^ (1.95)
Performance (% correct)	98.72^a^ (2.81)	71.00^b^ (18.96)	98.81^a^ (3.01)	66.30^b^ (21.19)	91.60^a^ (8.55)	83.56^b^ (13.94)	91.46^b^ (8.04)	92.42^a^ (7.35)	90.00^b^ (9.30)	88.29^a^ (8.74)
Reaction time (ms)	—	—	—	—	808.29^a^ (281.81)	814.34^a,b^ (279.47)	877.12^a,b^ (279.37)	841.22^a^ (267.35)	903.25^b^ (307.42)	1029.99^c^ (326.04)

*Note:* The working memory task included four trial types: neutral pictures presented under low load (low‐load neutral), negative pictures presented under low load (low‐load negative), neutral pictures presented under high load (high‐load neutral), and negative pictures presented under high load (high‐load negative). The recognition task included these same four previously presented (“old”) trial types, together with two additional trial types consisting of pictures that had not been presented during the working memory task (“new neutral” and “new negative”). The 400–800 ms time window was used to measure the LPP in the working memory task and the Old/New effect in the recognition task. Superscript letters denote pairwise differences at *p* < 0.05 within picture type, probed as a result of significant interaction effects. Significant main effects are not denoted in this table for brevity.

### 3.1 Working Memory Task

**FIGURE 2:**
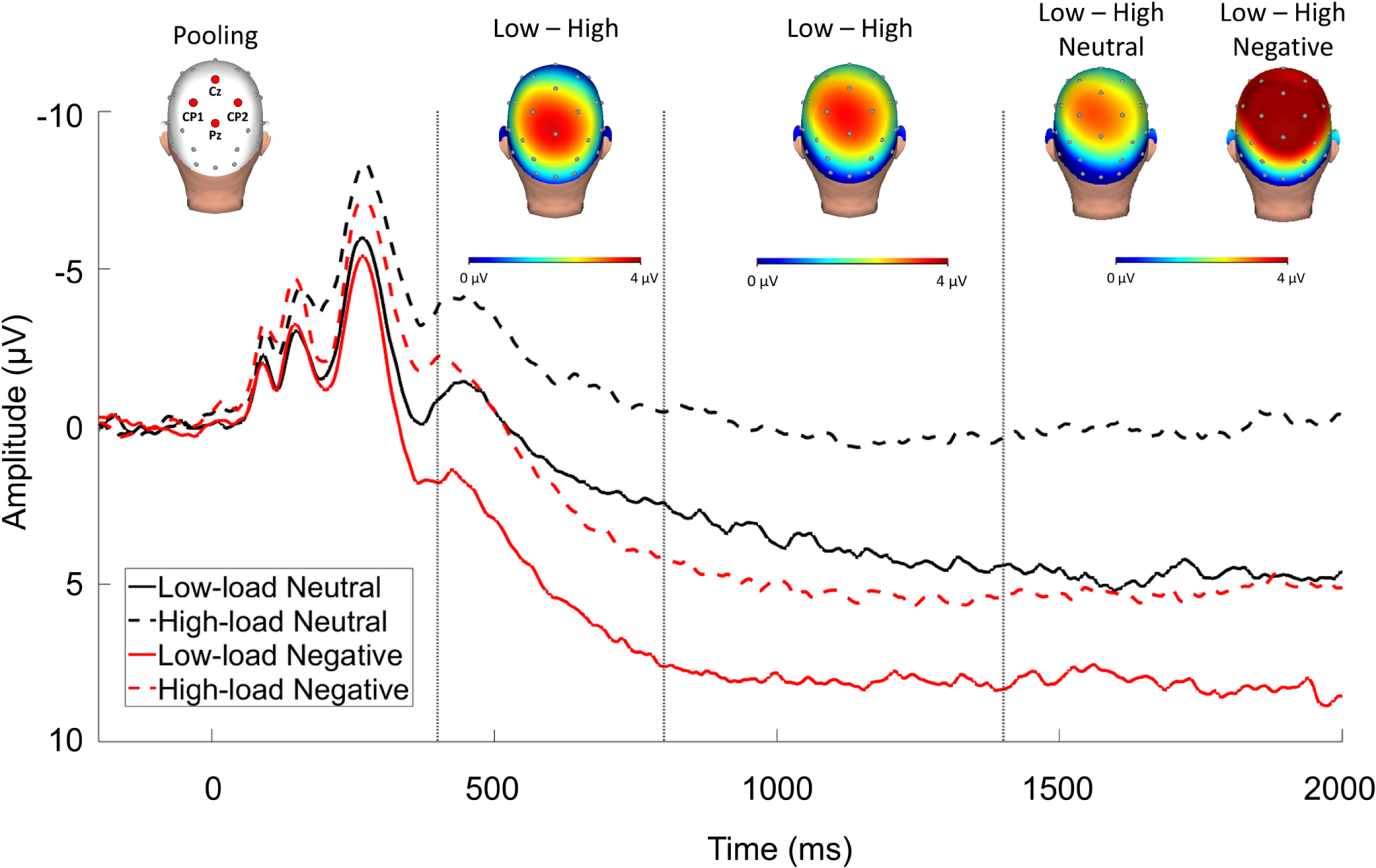
Working memory task: grand‐averaged waveforms at the centroparietal pooling where the LPP was scored. Dashed lines demarcate the 400–800, 800–1400, and 1400–2000 ms time windows, with topographic differences in amplitudes for low‐ versus high‐load pictures (neutral and negative collapsed together; 400–800 and 800–1400 ms windows) and low‐ versus high‐load pictures (neutral and negative separately; 1400–2000 ms window).

Figure 2 depicts grand‐averaged waveforms at the pooling where the LPP was scored, along with headmaps depicting the topography of the LPP for high‐ minus low‐load pictures, separately for each time window.

#### 3.1.1 LPP 400–800 ms (Working Memory Task)

Negative pictures elicited larger LPPs than neutral pictures, *F*(1, 72) = 82.93, *p* < 0.001, *η*_*p*_^*2*^ = 0.54. Pictures presented on high‐load trials elicited smaller LPPs compared to those presented on low‐load trials, *F*(1, 72) = 62.09, *p* < 0.001, *η*_*p*_^*2*^ = 0.46. There was no Condition × picture type interaction, *p* = 0.44.

#### 3.1.2 LPP 800–1400 ms (Working Memory Task)

Negative pictures elicited larger LPPs than neutral pictures, *F*(1, 72) = 113.49, *p* < 0.001, *η*_*p*_^*2*^ = 0.61. Pictures presented on high‐load trials elicited smaller LPPs compared to those presented on low‐load trials, *F*(1, 72) = 48.62, *p* < 0.001, *η*_*p*_^*2*^ = 0.40. There was no Condition × picture type interaction, *p* = 0.21.

#### 3.1.3 LPP 1400–2000 ms (Working Memory Task)

Negative pictures elicited larger LPPs than neutral pictures, *F*(1, 72) = 65.83, *p* < 0.001, *η*_*p*_^*2*^ = 0.48. Pictures presented on high‐load trials elicited smaller LPPs compared to those presented on low‐load trials, *F*(1, 72) = 45.45, *p* < 0.001, *η*_*p*_^*2*^ = 0.39. In addition, there was a significant Condition × picture type interaction, *F*(1, 72) = 5.84, *p* = 0.02, *η*_*p*_^*2*^ = 0.08, which indicated that working memory load more effectively reduced the LPP for neutral compared to negative pictures.

#### 3.1.4 Picture Ratings (Working Memory Task)

Negative pictures were rated as more arousing than neutral pictures, *F*(1, 72) = 289.36, *p* < 0.001, *η*_*p*_^*2*^ = 0.80. Arousal ratings did not differ for pictures presented on low‐load compared to high‐load trials, *p* = 0.48. There was no Condition × picture type interaction for arousal ratings, *p* = 0.64.

Negative pictures were rated as more unpleasant than neutral pictures, *F*(1, 72) = 431.42, *p* < 0.001, *η*_*p*_^*2*^ = 0.86. Valence ratings did not differ for pictures presented on low‐load compared to high‐load trials, *p* = 0.49. There was no Condition × picture type interaction for valence ratings, *p* = 0.69.

#### 3.1.5 Accuracy (Working Memory Task)

Participants recalled fewer letters on high‐load compared to low‐load trials, *F*(1, 72) = 182.14, *p* < 0.001, *η*_*p*_^*2*^ = 0.72 and fewer letters on trials with negative compared to neutral pictures, *F*(1, 72) = 12.31, *p* = 0.001, *η*_*p*_^*2*^ = 0.15. These effects were qualified by a Condition × picture type interaction, *F*(1, 72) = 15.23, *p* < 0.001, *η*_*p*_^*2*^ = 0.18, which indicated that the deleterious effect of working memory load on performance was larger for negative compared to neutral pictures.

### 3.2 Recognition Task

**FIGURE 3:**
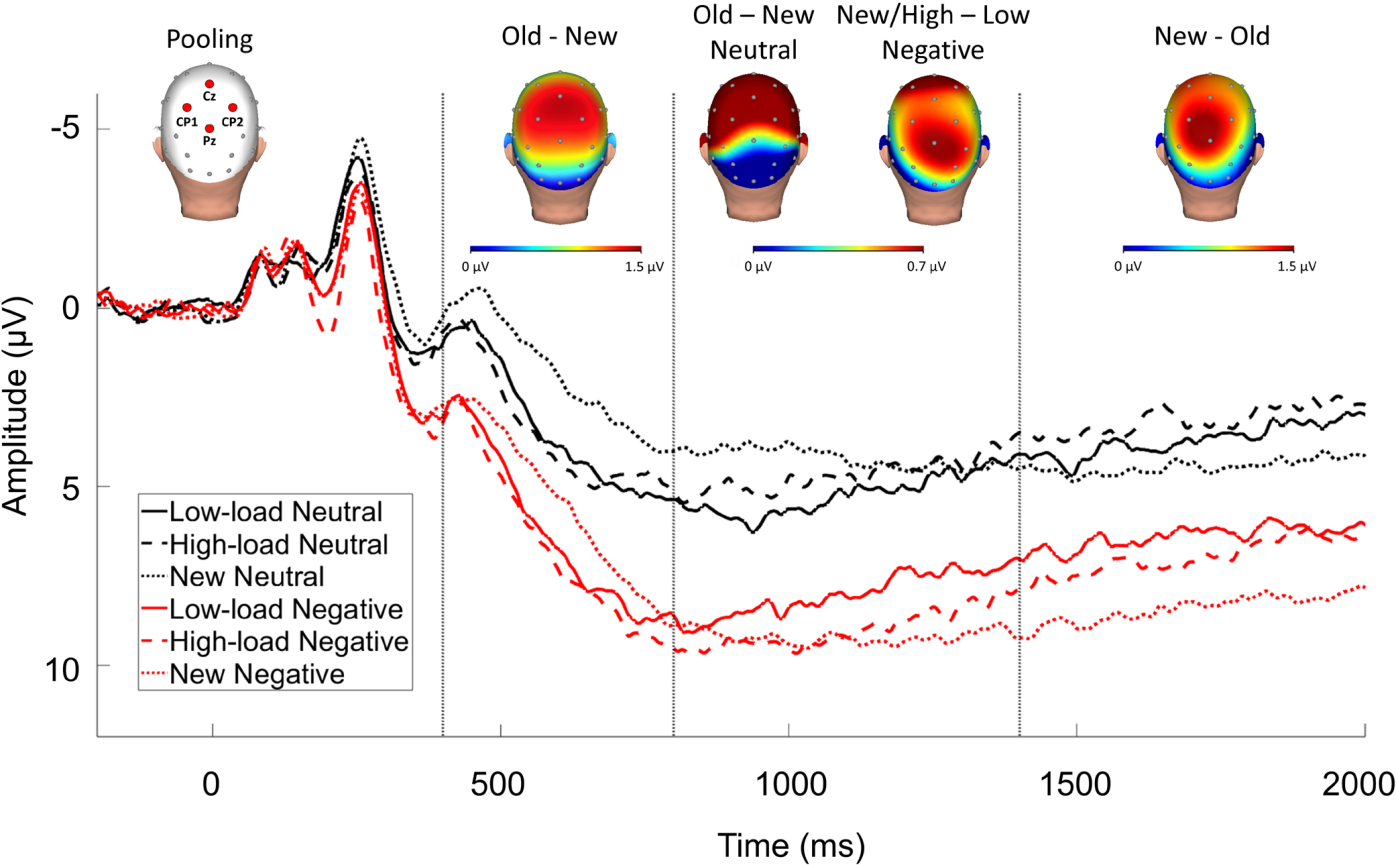
Recognition task: grand‐averaged waveforms at the centroparietal pooling where ERPs were scored. Dashed lines demarcate the 400–800, 800–1400, and 1400–2000 ms time windows, with topographic differences in amplitudes for old (low‐ and high‐load collapsed together) versus new (neutral and negative collapsed together; 400–800 ms), old versus new (neutral only, 800–1400 ms) and averaged high‐load and new versus low‐load (negative only, 800–1400 ms), and new versus old (1400–2000 ms).

Figure 3 presents grand‐averaged waveforms at the pooling where the Old/New and LPP positivities were scored, along with headmaps illustrating the main and interaction effects found in the subsequent analyses.

#### 3.2.1 Old/New Effect 400–800 ms (Recognition Task)

Negative compared to neutral pictures elicited larger amplitudes, *F*(1, 72) = 114.29, *p* < 0.001, *η*_*p*_^*2*^ = 0.61. A main effect of condition revealed an old‐new effect, *F*(2, 144) = 18.30, *p* < 0.001, *η*_*p*_^*2*^ = 0.20: Previously seen pictures elicited larger amplitudes than new pictures [high‐load > new, *t*(72) = 5.64, *p*_*adj*_ < 0.001; low‐load > new, *t*(72) = 4.85, *p*_*adj*_ < 0.001; low‐load versus high‐load, *p*_*adj*_ = 0.33; Table 1]. There was no Condition × picture type interaction, *p* = 0.29.

#### 3.2.2 LPP 800–1400 ms (Recognition Task)

Negative compared to neutral pictures elicited larger amplitudes, *F*(1, 72) = 140.14, *p* < 0.001, *η*_*p*_^*2*^ = 0.66. There was no main effect of condition, *p* = 0.79, however, there was a significant Condition × picture type interaction, *F*(2, 144) = 8.80, *p* < 0.001, *η*_*p*_^*2*^ = 0.11. Follow‐up tests revealed a significant effect of condition for each of negative, *F*(2, 144) = 4.03, *p* = 0.02, *η*_*p*_^*2*^ = 0.05 and neutral, *F*(2, 144) = 3.22, *p* = 0.04, *η*_*p*_^*2*^ = 0.04 pictures. Follow‐up tests comparing condition amplitudes separately for each picture type revealed a reversal effect for negative pictures: Old pictures previously presented on low‐load trials now elicited *smaller* LPPs than new negative pictures, *t*(72) = 2.76, *p*_adj_ = 0.02. However, amplitudes did not differ for high‐load negative compared to new negative trials, *p*_adj_ = 0.65 (i.e., low‐load negative< high‐load negative = new negative). Negative pictures previously presented on high‐load trials elicited larger amplitudes than negative pictures that had been presented on low‐load trials at a trend level, *t*(72) = 2.12, *p*_adj_ = 0.056. On the other hand, a reversal effect was not observed for neutral pictures: New pictures continued to elicit smaller LPPs than those that had been presented on low‐load trials *t*(72) =2.63, *p*_adj_ = 0.03, while pictures presented on high‐load trials were comparable to new neutral pictures, *p*_adj_ = 0.23 (low‐load neutral versus high‐load neutral, *p*_adj_ = 0.23).

#### 3.2.3 LPP 1400–2000 ms (Recognition Task)

Negative compared to neutral pictures elicited larger amplitudes, *F*(1, 72) = 97.74, *p* < 0.001, *η*_*p*_^*2*^ = 0.58. There was a main effect of condition, *F*(2, 144) = 9.73, *p* < 0.001, *η*_*p*_^*2*^ = 0.12, which indicated that new pictures elicited larger amplitudes than previously‐seen pictures [new> low‐load, *t*(72) = 3.63, *p*_adj_ < 0.001; new> high‐load, *t*(72) = 4.12, *p*_adj_ < 0.001; low‐load versus high‐load, *p*_adj_ = 0.98]. There was no Condition × picture type interaction, *p* = 0.13.

#### 3.2.4 Picture Ratings (Recognition Task)

Negative pictures were rated as more arousing than neutral pictures, *F*(1, 71) = 207.65, *p* < 0.001, *η*_*p*_^*2*^ = 0.74. The effect of condition did not reach significance, *p* = 0.16; however, there was a significant Condition × picture type interaction, *F*(2, 142) = 8.50, *p* < 0.001, *η*_*p*_^*2*^ = 0.11. There was a significant effect of condition for negative pictures, *F*(2, 142) = 6.07, *p* = 0.003, *η*_*p*_^*2*^ = 0.08 and neutral pictures, *F*(2, 142) = 3.66, *p* = 0.03, *η*_*p*_^*2*^ = 0.05. Follow‐up tests showed that negative pictures previously presented on high‐load trials were rated as more arousing compared to those presented on low‐load trials, *t*(71) = 3.44, *p*_adj_ = 0.003. In addition, new negative pictures were rated as more arousing than old negative pictures presented on low‐load trials, *t*(71) = 2.77, *p*_adj_ = 0.01, whereas ratings did not differ for new negative versus high‐load negative, *p*_adj_ = 0.63. On the other hand, for neutral pictures, new pictures, *t*(71) = 2.35, *p*_adj_ = 0.04 and neutral pictures previously presented on high‐load trials, *t*(71) = 2.26, *p*_adj_ = 0.04 were rated as *less* arousing compared to those previously presented on low‐load trials (new pictures versus high‐load pictures, *p*_adj_ = 0.53).

Negative pictures were rated as more unpleasant than neutral pictures, *F*(1, 71) = 373.72, *p* < 0.001, *η*_*p*_^*2*^ = 0.84. The effect of condition did not reach significance, *p* = 0.12; however, there was a significant Condition × picture type interaction, *F*(2, 142) = 4.71, *p* = 0.01, *η*_*p*_^*2*^ = 0.06. The effect of condition was significant for neutral pictures, *F*(2, 142) = 6.49, *p* = 0.002, *η*_*p*_^*2*^ = 0.08, but not negative pictures, *p* = 0.44. Follow‐up tests showed that new neutral pictures were rated as more pleasant than neutral pictures previously presented on low‐load trials, *t*(71) = 3.48, *p*_adj_ = 0.003. Neutral pictures previously presented on high‐load trials were rated somewhere between those presented on low‐load trials and new neutral pictures, but did not differ from either (high‐load neutral versus new neutral, *p*_adj_ = 0.09; high‐load neutral versus low‐load neutral, *p*_adj_ = 0.08).

#### 3.2.5 Accuracy (Recognition Task)

Accuracy was higher for negative compared to neutral pictures, *F*(1, 72) = 4.91, *p* = 0.03, *η*_*p*_^*2*^ = 0.06. There was also a main effect of condition, *F*(1.77,127.28) = 8.97, *p* < 0.001, *η*_*p*_^*2*^ = 0.11 that was qualified by an Condition × picture type interaction, *F*(1.73,124.72) = 25.73, *p* < 0.001, *η*_*p*_^*2*^ = 0.26. The effect of condition was significant for negative pictures, *F*(2, 144) = 5.35, *p* = 0.006, *η*_*p*_^*2*^ = 0.07 and for neutral pictures, *F*(1.70, 122.32) = 17.81, *p* < 0.001, *η*_*p*_^*2*^ = 0.20. Participants were less accurate for negative pictures previously presented under high‐ compared to low‐load, *t*(72) = 2.20, *p*_adj_ = 0.04. Accuracy was also lower for new compared to low‐load negative pictures, *t*(72) = 3.18, *p*_adj_ = 0.006, but did not differ for high‐load versus new pictures, *p*_adj_ = 0.22 (i.e., high‐load negative = new negative< low‐load negative). Follow‐up tests showed that participants were also less accurate for neutral pictures presented under high‐load compared to low‐load, *t*(72) = 5.85, *p*_adj_ < 0.001, and new pictures, *t*(72) = 4.30, *p*_adj_ < 0.001, whereas accuracy did not differ for low‐load compared to new pictures, *p*_adj_ = 0.92 (i.e., high‐load neutral < low‐load neutral = new neutral).

#### 3.2.6 Reaction Time (Recognition Task)

Participants were faster to respond to neutral compared to negative pictures, *F*(1, 72) = 53.42, *p* < 0.001, *η*_*p*_^*2*^ = 0.43. A main effect of condition, *F*(2, 144) = 25.04, *p* < 0.001, *η*_*p*_^*2*^ = 0.26, indicated that participants were slower to respond to pictures previously presented on high‐load compared to low‐load trials, *t*(72) = 2.11, *p*_adj_ = 0.04. In addition, participants were slower to respond to new compared to previously seen pictures [new > low‐load, *t*(72) = 6.63, *p*_adj_ < 0.001; new > high‐load, *t*(72) = 4.58, *p*_adj_ < 0.001]. The main effect of condition was qualified by a significant Condition × picture type interaction, *F*(2, 144) = 7.28, *p* = 0.001, *η*_*p*_^*2*^ = 0.09. Follow‐up tests revealed a significant effect of condition for negative pictures, *F*(2, 144) = 38.13, *p* < 0.001, *η*_*p*_^*2*^ = 0.35 and for neutral pictures, *F*(2, 144) = 4.03, *p* = 0.02, *η*_*p*_^*2*^ = 0.05. Results for negative pictures were in line with the overall effect of condition: Participants were slower to respond to negative pictures presented on high‐load compared to low‐load trials, *t*(72) = 2.95, *p*_adj_ = 0.004. In addition, participants were slower to respond to new than previously seen negative pictures [new negative > low‐load negative, *t*(72) = 8.64, *p*_adj_ < 0.001; new negative > high‐load negative, *t*(72) = 5.47, *p*_adj_ < 0.001]. Similarly, participants were slower to respond to new versus low‐load neutral pictures, *t*(72) = 2.60, *p*_adj_ = 0.03, and high‐load neutral pictures at a trend level, *t*(72) = 2.14, *p*_adj_ = 0.054. However, there was no difference in reaction time for neutral pictures previously presented on high‐load compared to low‐load trials, *p*_adj_ = 0.81.

## 4 Discussion

In our previous work (MacNamara et al. 2011, 2012, 2019; MacNamara and Proudfit 2014) we found that attention toward task‐irrelevant negative and neutral pictures, as measured by the LPP, was reduced when participants were asked to perform a demanding cognitive task (i.e., high versus low working memory load). Here, we examined whether initial presentation of pictures under working memory load would affect the subsequent processing of these pictures in a recognition task.

Within‐subjects effects for the working memory task were in line with those observed in our prior work. That is, in addition to the expected picture type effect (negative > neutral), we observed smaller LPPs for pictures presented under high compared to low working memory load, suggesting reduced salience of task‐irrelevant pictures when task demands are high (MacNamara et al. 2011, 2012, 2019; MacNamara and Proudfit 2014). We also found an interaction effect of picture Type X working memory load during the later time window (1400–2000 ms), where higher working memory attenuated the LPP more to neutral, compared to negative pictures. Considering that the LPP is known to index motivational salience, the moderating effect of working memory load may have been more evident for neutral pictures because they competed less for attentional resources with the primary working memory task compared to negative pictures. Our accuracy findings support this speculation, where the deleterious effect of working memory load on task performance was more pronounced for negative pictures (MacNamara et al. 2011, 2019; MacNamara and Proudfit 2014). We did not observe an effect of working memory load for valence or arousal ratings, in line with prior work using a similar task design (Adamczyk et al. 2022), suggesting that the modulatory role of working memory load in processing task‐irrelevant pictures may not be detectable at the subjective level.

In the recognition task, amplitudes were more positive for negative compared to neutral pictures across all three time windows (Codispoti et al. 2006). During the early time window (400–800 ms), we observed larger amplitudes for old compared to new pictures, in line with the parietal old/new effect (Rugg et al. 1996). Of note, although repetition work from the LPP literature has found evidence of *smaller* LPPs for old compared to new pictures in the 400–800 ms window, this work has used passive picture viewing tasks and massed repetition (e.g., 60 repetitions or more of each picture), distinguishing it from the recognition literature (for a review, see Ferrari et al. 2017).

In the 800–1400 ms window, we observed a reversal effect (Rugg et al. 1996) for negative pictures only: Larger amplitudes for *new* negative pictures compared to those previously presented on low‐load trials with no differences observed between new and high‐load negative pictures. Neutral pictures did not show this effect; instead, new neutral pictures continued to elicit smaller amplitudes than neutral pictures previously presented on low‐load trials (with no differences observed between new and high‐load neutral pictures), suggesting that the reversal effect may be delayed for neutral pictures, beginning in the 1400–2000 ms and not the 800–1400 ms window. Similarly, Lavoie and O'Connor (2013) observed an old/new effect for negative pictures that emerged at 500–700 ms, disappeared at 700–1000 ms, then reversed by 1000–1400 ms post‐stimulus onset. In contrast, for neutral pictures, the old/new effect persisted through the 700–1000 ms window and trended toward reversal by 1000–1400 ms. Though overlap between the LPP and LPN during the later time window precludes definitive conclusions regarding underlying cognitive processes, one potential explanation for our results may be that the negative pictures may carry more motivational salience, facilitating an earlier retrieval monitoring process indexed by the LPN. This negativity may dampen the relative positivity associated with previously seen pictures during later stages of processing, emerging as an earlier flip from the old/new effect (old > new) to the reversal effect (old < new). As such, both working memory load and emotional salience may shape the temporal pattern of recognition‐related processing. But eventually—that is, by the 1400–2000 ms window—reversal effects were evident for both negative and neutral pictures (i.e., new > old).

Critically, our results suggest that negative pictures viewed under high working memory load may, after an initial determination of whether the picture is old or new (i.e., the old/new effect observed during 400–800 ms), undergo a subsequent step in which they receive more elaborated processing, as if they were never seen before. This may be due to less fine‐grained encoding during the initial working memory task for negative pictures presented under high‐load (Naveh‐Benjamin et al. 1998). That is, though the LPP does not directly index encoding, it provides a measure of attentional and motivational processes that may facilitate encoding. This interpretation is consistent with a recent framing of the LPP as a measure of operations that support the integration of motivationally salient stimuli into contextual memory (Fields 2023). For example, when pictures are sorted into those that are later remembered versus forgotten, those that are later remembered elicited more positive LPPs at their initial presentation (Fabiani et al. 1986; Paller et al. 1987; Rugg and Allan 1995). Therefore, given that larger LPPs have been associated with more successful encoding, one plausible interpretation of the current results is that high‐load pictures may have been encoded less efficiently than low‐load pictures during the working memory task (i.e., smaller LPPs for high‐load pictures versus low‐load pictures). Further, prior work found a “subsequent memory effect” that was potentiated for emotional compared to neutral pictures (Dolcos and Cabeza 2002), which dovetails with the current findings during the 800–1400 ms time window showing that high‐load pictures elicited comparable LPPs to new pictures, but marginally larger LPPs than low‐load pictures—only for negative pictures.

A complementary explanation for our results is that high working memory load may have also interfered with habituation to stimuli. Smaller LPPs in the late time window for old compared to new pictures suggest that participants may habituate to previously seen stimuli, in line with prior work that has examined the effects of repetition on the LPP (Codispoti et al. 2006). However, habituation relies on the availability and allocation of processing resources and may therefore vary depending on the circumstances under which pictures are viewed. For example, prior work by Paul et al. (2016) found that participants had smaller LPPs to negative pictures previously viewed under instructions to “attend” rather than “distract” (see also Thiruchselvam et al. 2011), suggesting that greater initial attentional engagement may reduce subsequent emotional reactivity to those stimuli. Therefore, after an initial recognition stage—that is, once a reversal effect is observed, in the middle time window—larger LPPs to negative pictures initially presented under high compared to low working memory load may reflect incomplete habituation resulting from less complete initial processing under higher attentional demands.

The possibility that limited encoding and/or habituation may have resulted in participants processing high‐load pictures like new pictures is also supported by behavioral data collected during the recognition task. That is, participants rated high‐load pictures to be similarly arousing to new pictures for both negative and neutral pictures—that is, high working memory load and new pictures elicited more arousing ratings for negative pictures and less arousing (and less unpleasant) ratings for neutral pictures. This pattern is in line with our ERP findings observed during the 800–1400 ms time window, where high‐load negative pictures elicited comparable LPP amplitudes to new negative pictures. Previous work has shown that novel emotional (but not neutral) pictures are perceived as more arousing compared to previously seen pictures, possibly due to habituation reducing emotional responding (Codispoti et al. 2006). Thus, it may be that high working memory load impaired habituation of response to negative pictures, leading to responses that resembled those elicited by new negative pictures when they were encountered again later on.

Moreover, participants were more likely to incorrectly judge a high‐load picture to not have been seen before than a low‐load picture, implying that pictures viewed under high working memory load may not have been processed enough to have been stored accurately in long term memory. Participants were also slower to respond to high‐load pictures than low‐load pictures, possibly because high‐load pictures were less robustly encoded, requiring longer evaluation during retrieval. Importantly, this pattern was clearest for negative pictures, aligning with our ERP findings. Together, these findings further bolster the interpretation that pictures previously viewed under high working memory load may have been experienced as less familiar, compared to those viewed under low working memory load, when encountered again during the recognition task.

The current study examined the effects of cognitive load on emotional picture processing over time and found that these effects may be reversed upon subsequent presentation of images, leading to *increased* LPPs/attention toward negative stimuli. In other words, pictures viewed under high working memory load may be experienced as less familiar and more emotionally salient when viewed again, possibly because they were insufficiently processed during initial presentation. This may provide a partial explanation as to why disengaged emotion regulation strategies may be less effective in the long term (MacNamara 2025; Sheppes et al. 2014).

Overall, the current findings suggest that high working memory load at the time of initial picture presentation may affect subsequent encounters with stimuli, with potentially differing effects for negative and neutral pictures. Though working memory load attenuated immediate responses to task‐irrelevant pictures, this resulted in later patterns of neural and behavioral responding consistent with reduced familiarity for both picture types. The overall direction of effects differed for neutral compared to negative pictures, but pictures that had been presented under high working memory resembled new pictures in the responses they elicited across both picture types. For negative stimuli, results may elucidate a mechanism through which disengaged emotion regulation strategies may fail to attenuate and may even increase emotional responses to stimuli when encountered again outside of the initial regulation context.

## Author Contributions

**Ha Jeong Park:** formal analysis, writing – original draft, writing – review and editing. **T. Bryan Jackson:** formal analysis, writing – original draft, writing – review and editing. **Annmarie MacNamara:** conceptualization, funding acquisition, writing – review and editing, methodology, supervision.

## Funding

This work was supported by National Institute of Mental Health grants, K23MH105553 (to AM) and R01MH125083 (to AM) during the preparation of this manuscript.

## Conflicts of Interest

The authors declare no conflicts of interest.

## Data Availability

The data that support the findings of this study are openly available in Open Science Framework at https://osf.io/cxgzk.

## Endnotes

^1^
Old negative list 1 (high‐load in version 1; low‐load in version 2): 2122441201 1300 2120 3059 3100 3181 3266 3550 6243 6260 6540 6562 6570 6825 6832 9042 9050 9075 9250 9252 9253 9413 9490 9520 9582 9621 9902 9911; list 2 (low‐load in version 1; high‐load in version 2): 2182322462502511052 1202 1302 2811 3103 3216 3350 3530 6263 6370 6510 6520 6530 9265 9332 9405 9410 9420 9424 9433 9599 9611 9900 9920 9921. Old neutral list 1 (high‐load in version 1; low‐load in version 2): 1031361391851953542026 2230 2385 2400 2411 2446 2516 5500 5740 6150 7009 7014 7017 7019 7026 7033 7040 7041 7090 7130 7217 7490 7705 7950; list 2 (low‐load in version 1; high‐load in version 2): 1071081381591653392039 2104 2441 2495 2840 5120 5534 7003 7018 7020 7030 7032 7037 7060 7080 7110 7140 7180 7224 7493 7496 7547 7550 7920. New negative list: 2112142152162242312382472482523213251114 1120 1205 1304 1931 2800 3015 3016 3051 3110 3130 3160 3168 3180 3195 3213 3225 3230 3280 3300 3301 6212 6230 6250 6312 6313 6315 6350 6560 9040 9412 9414 9419 9421 9423 9425 9428 9435 9491 9561 9584 9622 9635.19901 9904 9909 9910 9912; New neutral list: 1091221231241401411481671801881911963663731450 1670 2038 2102 2107 2190 2191 2384 2393 2396 2397 2493 2512 2514 2595 2745.15531 7000 7002 7004 7006 7010 7012 7021 7031 7035 7036 7042 7050 7055 7056 7078 7081 7150 7175 7211 7233 7234 7235 7491 7500 7504 7546 7560 7595 7700. Numbers < 1000 refer to pictures from the EmoPics stimulus set (Wessa et al. 2010); numbers > 1000 refer to pictures from the IAPS stimulus set (Lang et al. 2005).

^2^
Picture assignment to high or low working memory load conditions was randomized for each participant, but picture assignment to old/new was not. All participants viewed the same set of old/new pictures. To confirm that picture lists assigned to old versus new conditions was not confounded with picture arousal or valence, we collapsed list 1 and list 2 used during the working memory task (old pictures) and subjected normed arousal and valence ratings (Lang et al. 2005; Wessa et al. 2010) separately to 2 (condition: old, new) × 2 (picture type: negative, neutral) ANOVAs. The only significant effects found were for picture type: arousal: *F*(1, 236) = 1359.72, *p* < 0.001, *η*_*p*_^*2*^ = 0.85; valence: *F*(1, 236) = 1198.40, *p* < 0.001, *η*_*p*_^*2*^ = 0.84. All other *ps* > 0.19. Normed valence and arousal ratings for pictures were as follows: old neutral: valence, *M* = 4.90, SD = 0.38, arousal, *M* = 3.11, SD = 0.56; old negative, valence, *M* = 2.55, SD = 0.65, arousal, *M* = 6.05, SD = 0.60; new neutral, valence, *M* = 5.01, SD = 0.45, arousal, *M* = 3.10, SD = 0.64; new negative, valence, *M* = 2.47, SD = 0.65, arousal, *M* = 6.05, SD = 0.68.

^3^
The percentage of trials that survived artifact rejection per condition in each task was as follows: working memory task—low neutral *M* = 97.12 (SD = 0.07), high neutral *M* = 97.30 (SD = 0.08), low negative *M* = 97.53 (SD = 0.07), high negative *M* = 97.39 (SD = 0.08); recognition task (correct trials only)—low neutral *M* = 98.42 (SD = 0.03), high neutral *M* = 97.74 (SD = 0.05), new neutral *M* = 97.77 (SD = 0.04), low negative *M* = 98.44 (SD = 0.03), high negative 97.89 (SD = 0.04) and new negative *M* = 98.11 (SD = 0.04).

^4^
Although recognition memory is often presented in terms of hits and correct rejections, here we wanted to compare accuracy across different levels of WM load (i.e., hits) with accuracy for new pictures (i.e., correct rejections). Therefore, we analyzed hits and correct rejections in a single ANOVA (Herron et al. 2003).
